# Acupuncture for treating whiplash-associated disorder: A systematic review and meta-analysis protocol

**DOI:** 10.1097/MD.0000000000012654

**Published:** 2018-10-12

**Authors:** Seunghoon Lee, Dae-Hyun Jo, Kun Hyung Kim

**Affiliations:** a Department of Acupuncture & Moxibustion Medicine; b Department of Acupuncture & Moxibustion Medicine, Graduate School, Kyung Hee University, Seoul; c Department of Acupuncture & Moxibustion, School of Korean Medicine, Pusan National University, Yangsan, South Korea.

**Keywords:** acupuncture, protocol, systematic review, traffic accident, whiplash injury, whiplash-associated disorder

## Abstract

**Background:**

This review aims to evaluate the effectiveness and safety of acupuncture treatment for patients with whiplash-associated disorder (WAD).

**Methods:**

We will search the following databases from their inception to October 2018: MEDLINE, Embase, the Cochrane Central Register of Controlled Trials, the Cumulative Index to Nursing and Allied Health Literature, the Allied and Complementary Medicine Database, 1 Chinese database (China National Knowledge Infrastructure), 1 Japanese database (Japan Science and Technology Information Aggregator, Electronic), and 5 Korean databases (KoreaMed, Research Information Service System, Korean Studies Information Service System, Database Periodical Information Academic, and Oriental Medicine Advanced Searching Integrated System). All randomized controlled trials of acupuncture for WAD will be considered for inclusion without language restrictions. The risk of bias will be assessed using the Cochrane risk of bias tool. The mean difference or standard mean difference for continuous data and risk ratio for dichotomous data will be calculated with 95% confidence intervals.

**Dissemination:**

The results of this review will be disseminated through peer-reviewed journal articles or conference presentations, and may provide important guidance for clinicians and patients regarding the use of acupuncture treatment for treating WAD.

Trial registration number: PROSPERO 2018: CRD42018106964.

## Abstract 2

Supplemental Digital Content is available in the text

## 1 Introduction

### 1.1 Description of the condition

The term of whiplash associated disorder (WAD) was introduced by the Quebec Task Force (QTF) in 1995 to reflect the spectrum of clinical symptoms following bony or soft-tissue injuries resulting largely from motor vehicle collisions.^[1]^ The predominant symptoms of WAD include neck pain and stiffness, and other pain in the back, shoulder, and temporomandibular joints. Apart from pain, WAD symptoms include dizziness, visual disturbance, fatigue, sleep disturbance, anxiety, depression, memory difficulties, and psychological distress.^[2–4]^ The incidence of WAD is estimated to be 300 per 100,000 inhabitants, although these rates differ between countries.^[2]^ The associated annual costs after road accidents in 2016 is estimated to be more than £35 billion in the United Kingdom^[5]^ and more than US $21 billion in the South Korea, which accounts for approximately 1.4% of the gross domestic product of South Korea;^[6]^ these estimates are consistent even when the method of calculation is altered to include output, medical care, damage to property, and police cost. Several prospective studies have report that most recovery takes place up to 3 months following the initial injury,^[7,8]^ and, therefore, proper treatment management in the acute and subacute stages is important to prevent the development of chronic conditions.^[2]^

### 1.2 Description of the intervention

Acupuncture is defined as an intervention that stimulates specific points (e.g., traditional acupuncture points, myofascial trigger points, or tender points) using needles with various manipulations (e.g., manual or electrical stimulation). It has mainly been used for the treatment of musculoskeletal diseases such as neck, back, or knee pain, and its effectiveness and safety have been supported by many rigorous clinical trials.^[9]^ Recently, the application of acupuncture therapy has been extended to include treatment for psychological symptoms related to posttraumatic stress disorder, as well as comorbidities for chronic pain, such as insomnia, depression, or anxiety.^[10,11]^

### 1.3 How the intervention might work

The mechanism of acupuncture treatment for pain and psychological symptoms remains unclear. Some studies have proposed mechanisms of acupuncture analgesia according to: local effect mediated by adenosine A1 receptors^[12,13]^ or myofascial trigger point inactivation;^[14]^ segmental effect based on the gate-control theory of pain;^[15]^ and a general effect through descending inhibitory pain control by serotonin and noradrenaline.^[16]^ Moreover, the mechanism of acupuncture for psychological symptoms in chronic pain or mental illness is thought to involve modulation of opioid peptides and monoamines, such as noradrenaline, serotonin, or dopamine in the brain.^[17]^

### 1.4 Why it is important to perform this review

The value of acupuncture treatment for treating WAD is controversial based on the limited systematic reviews and clinical practice guidelines available. One systematic review reported that the evidence of acupuncture for WAD was inconclusive due to limited data.^[18]^ Updated evidence suggests that acupuncture may not be effective in treating neck pain induced by WAD because acupuncture treatment has not been shown to reduce neck pain at a level that was clinically significant. However, the trials included in this review only compared acupuncture therapy with sham acupuncture, which is known to have more than a placebo effect and not be valid as an inert placebo control.^[19,20]^ Moreover, they did not conduct a systematic search, and they only evaluated literature published in English.^[20,21]^ Therefore, it is worth conducting a systematic review that includes comparative effectiveness trials, as well as sham-controlled trials, with an up-to-date systematic search to determine whether acupuncture is an effective treatment option for WAD.

### 1.5 Objective

The objective of this systematic review is to evaluate the benefits and harms of acupuncture therapy for patients with WAD in comparison to those who received with no treatment, sham acupuncture, routine/usual care, conventional medicine, or other active treatments.

## 2 Methods

### 2.1 Study registration

The protocol of review methods has been registered, prospectively (CRD42018106964; http://www.crd.york.ac.uk/ PROSPERO).

### 2.2 Criteria for including studies in this review

#### 2.2.1 Types of studies

Prospective randomized controlled trials (RCTs) of acupuncture treatment for WAD will be included in the review. Nonrandomized controlled trials, observational studies, qualitative studies, and laboratory studies will be excluded. Language will not be restricted for study eligibility.

#### 2.2.2 Types of participants

All patients who suffered from the any symptoms related to WAD, such as musculoskeletal pain, sensorimotor control disturbances, or psychological problems will be included. The diagnosis criteria and classification of WAD will not be limited.

#### 2.2.3 Types of interventions

Acupuncture treatment using needling with various types of stimulation (e.g., manual or electrical) on specific points (e.g., traditional acupuncture points, myofascial trigger points, or tender points) will be included. However, trials involving non-penetrating stimulation on specific points (e.g., acupressure, magnets, moxibustion, transcutaneous electrical nerve stimulation, or laser therapy) and penetrating stimulation with the insertion of medical materials (e.g., herbal acupuncture or thread embedding acupuncture) will not be included in the review.

The control intervention will be considered as no treatment/waiting list, sham acupuncture, and active treatment (e.g., medication or physiotherapy).^[22]^ However, trials in which acupuncture was compared with other forms of acupuncture or herbal medication will be excluded. When the acupuncture group received acupuncture and other active treatment simultaneously, only trials in which the same active treatment was administered to the both groups will be included.

#### 2.2.4 Types of outcome measures

The time frame of outcome measurements will be determined as a short-term (up to 12 weeks after injury) and a long-term outcome (more than 12 weeks after injury).

##### 2.2.4.1 Primary outcomes

1. Severity of pain: the measurement of relevant pain using any scale (e.g., visual analog scale (VAS) (0–100 mm or 0–10 cm) or numerical rating scale [NRS]) will be analyzed.
2. Function: relevant overall function and disability using any scale or range (e.g., neck disability index [NDI])

##### 2.2.4.2 Secondary outcomes

1. Quality of life (QoL): assessed using a validated scale (e.g., 36-item Short-Form [SF-36] or Euro-QoL)
2. Range of movement (ROM) of the neck
3. Psychological measurements
4. Clinical global improvement in symptoms
5. Adverse events related to acupuncture treatment

### 2.3 Search methods for identification of studies

#### 2.3.1 Electronics searches

The following 12 databases will be searched from inception to October 2018: MEDLINE (1946 to October Week 4 2018), Embase (1980 to October 4, 2018), the Cochrane Central Register of Controlled Trials (*The Cochrane Library*, 2018 Issue 10), the Cumulative Index to Nursing and Allied Health Literature (CINAHL, 1982 to October 2018), the Allied and Complementary Medicine Database (AMED, 1985 to October 2018), 1 Chinese database (China National Knowledge Infrastructure (CNKI)), 1 Japanese database (Japan Science and Technology Information Aggregator Electronic (J-STAGE)), and 5 Korean databases (KoreaMed, Research Information Service System (RISS), Korean Studies Information Service System (KISS), Database Periodical Information Academic (DBpia), and Oriental Medicine Advanced Searching Integrated System (OASIS)).

#### 2.3.2 Search for other resources

The WHO International Clinical Trials Registry Platform will be also searched for ongoing and recently completed studies. Bibliographic references in relevant publications will be manually searched to avoid missing eligible trials.

#### 2.3.3 Search strategy

The search terms will consist of 2 parts: WAD (e.g., whiplash, traffic, or neck injury) and acupuncture (e.g., acupuncture, electroacupuncture, or dry needling). The detailed search strategies for MEDLINE are presented in the online supplementary appendix 1.

### 2.4 Data collection and analysis

#### 2.4.1 Selection of studies

Two review authors (SL and KHK) will independently screen the titles and abstracts for potentially eligible studies identified by the searches. The authors will independently select and record their decisions according to predefined criteria on a standard eligibility form. If a disagreement between 2 reviewers for study selection cannot be resolved through discussion, a third reviewer will resolve the disagreement. The flow process of filtration will be summarized in a Preferred Reporting Items for Systematic Reviews and Meta-Analysis (PRISMA)-compliant flow chart (http://www.prisma-statement.org).

#### 2.4.2 Data extraction and management

Two review authors (SL and KHK) will independently extract data from the articles using a standard data extraction form (e.g., author, year of publication, country, study design, sample size, participants, condition, acupuncture intervention, control intervention, outcome measures, main results, and adverse events) after reading the full text of each article. Details of the acupuncture treatment and control interventions will be extracted based on the revised STandards for Reporting Interventions in Clinical Trials of Acupuncture (STRICTA) guidelines.^[23]^ Any disagreement regarding the extracted data will be resolved through discussion or consultation among the reviewers. When the data are insufficient or unclear, we will contact the first author or the corresponding author through e-mail or telephone to request additional information.

#### 2.4.3 Assessment of risk of bias

Two review authors (SL and KHK) will independently perform the quality assessment using the tool for assessing risk of bias based on the Cochrane Handbook for Systematic Reviews of Interventions.^[24]^ The following domains will be assessed: random sequence generation; allocation concealment; blinding of participants/personnel; blinding of outcome assessors; incomplete outcome data; selective outcome reporting; and other sources of bias (including factors that are likely to influence the results, such as extreme baseline imbalance of age, comorbidities, disease onset, or physical conditions). The risk of bias will be categorized into 3 levels: low, high, and unclear risk of bias. Any disagreement will be resolved through discussion or consultation among the reviewers.

#### 2.4.4 Measures of treatment effect

For continuous outcomes, the mean difference (MD) with 95% confidence intervals (CIs) will be presented. If the methods or scales measuring the treatment effect among the studies in the analysis are not the same, the standardized mean difference (SMD) will be used. For dichotomous outcomes, the risk ratio (RR) will be used to measure the treatment effect with 95% CIs. Ordinal data will be converted to dichotomous data when the data needs to be pooled. For example, global assessments which were graded as “recovery,” “markedly effective,” “effective,” and “ineffective” will be dichotomized into “improved” or “not improved.”

#### 2.4.5 Unit of analysis issues

When unit of analysis issues arise in the studies that assessed outcome variable repeatedly (at more than one time point), we will categorized the assessments into 3 different measurement time frames after the traffic accident: acute stage (until 4 weeks), subacute stage (until 12 weeks), and chronic stage (over 12 weeks). If more than 2 assessments are reported in the same time frame, only the last assessment in the time frame will be chosen for analysis.

#### 2.4.6 Dealing with missing data

For missing or incomplete data, we will attempt to contact the original study authors to request the missing data. If the additional data cannot be obtained, only the available data will be analyzed, and the potential impact of the missing data will be addressed in the discussion.

#### 2.4.7 Assessment of heterogeneity

Heterogeneity will be assessed preferentially by visual inspection of the forest plot, and a χ^2^ test with a significance level of *P* < .10 will define the presentation of heterogeneity. Additionally, *I*^2^ statistic will be assessed to quantify the inconsistencies among the studies, with a value of more than 50% indicating a meaningful heterogeneity. *I*^2^ of 0% to 40% may be unimportant, 30% to 60% may be moderate, 50% to 90% may be substantial, and 75% to 100% may be considerable heterogeneity.^[24]^

#### 2.4.8 Assessment of reporting biases

Funnel plot will be used to detect reporting bias when more than 10 studies are available,^[24]^ and Egger's regression method will be used to determine funnel plot asymmetry.^[25]^

#### 2.4.9 Data synthesis

The meta-analysis using Review Manager software (RevMan, version 5.3 for Windows; the Nordic Cochrane Centre, Copenhagen, Denmark) will be used. A random effects model will be used to calculate the pooled effect estimates, because substantial clinical heterogeneity is expected across the included studies in this review. If considerable heterogeneity (*I*^2^ ≥ 75%) cannot be explained by the clinical and methodological diversity, the data will not be pooled.^[24]^ When a study has more than 2 acupuncture groups with different stimulation styles (e.g., manual or electrical stimulation) or points (e.g., local or distal points), meta-analysis will be conducted in careful consideration of whether the data of the different acupuncture groups will be combined into one merged acupuncture group.^[26]^

#### 2.4.10 Subgroup analysis and investigation of heterogeneity

When sufficient numbers of studies are available, subgroup analysis will be performed to identify the heterogeneity among studies according to the following:

1. Type of acupuncture stimulation (e.g., manual versus electrical needle stimulation)
2. Type of control (e.g., no treatment/waitlist, sham acupuncture, routine/usual care, conventional medicine, or other active treatments)
3. Severity of signs and symptoms assessed by the QTF grading system (e.g., grade I, II or III, IV).

#### 2.4.11 Sensitivity analysis

Where appropriate, sensitivity analysis will be conducted to evaluate whether the results are robust in the review according to the following:

1. Methodological qualities (e.g., whether random sequence generation, allocation concealment, and assessor blinding are adequately conducted or not)
2. Statistical method (random-effects model vs fixed-effects model)

#### 2.4.12 Summary of evidence

The results of the main outcomes (primary outcomes and adverse events) will be summarized in the “Summary of findings” tables. The quality of evidence in the main outcomes will be evaluated using the Grading of Recommendations Assessment, Development and Evaluation (GRADE) software with respect to the following aspects: limitations in the design and implementation; indirectness of evidence; unexplained heterogeneity or inconsistency of results; imprecision of results; and high probability of publication bias. The quality of evidence will be categorized into 4 levels: high, moderate, low, and very low quality.^[24]^

### 2.5 Ethics and dissemination

Ethical approval is not necessary as this study is a systematic review. The results of this review will be disseminated through peer-reviewed journal articles and conference presentations.

## 3 Discussion

This is the study protocol of a systematic review and meta-analysis for the use of acupuncture therapy to treat patients with WAD. A recent systematic review^[20]^ and a current clinical guideline^[21]^ suggested that there was not sufficient data to determine the effectiveness of needle acupuncture for WAD; additionally, they reported that electroacupuncture was not likely to be effective and it should not be considered as a form of treatment for neck pain. However, these reviews are based on studies that included only trials with sham needling, which may be as powerful as verum acupuncture although the value of acupuncture could be evaluated comparing with standard treatment as in a comparative effectiveness trials.^[19]^ Moreover, it is known that language restrictions can impact the estimates of effectiveness in complementary and alternative medicine interventions.^[27]^ However, these studies only searched English databases, and did not include studies in the Korean or Chinese literature. A recent review emphasized that Korean studies should be considered in a systematic review for acupuncture treatment to avoid potential language bias.^[28]^

Therefore, we will search all relevant literature, without any language restrictions, in the Korean, Chinese, and Japanese databases to include any relevant trials of acupuncture for treating patients with WAD. The results of this systematic review will provide a summary of the current evidence on the effectiveness and safety of acupuncture for WAD. This evidence will be a useful resource to patients, practitioners, and health policy-makers who want to consider acupuncture for WAD as a primary form of treatment or an adjuvant therapy to conventional treatments.

## Author contributions

**Conceptualization:** Seunghoon Lee, Kun Hyung Kim

**Funding acquisition:** Seunghoon Lee

**Methodology and project administration:** Seunghoon Lee, Kun Hyung Kim, Dae-Hyun Jo

**Writing – original draft:** Seunghoon Lee

**Writing – review & editing:** Kun Hyung Kim, Dae-Hyun Jo

## Supplementary Material

Supplemental Digital Content
