# Supplementary material for: Acupuncture for treating whiplash-associated disorder: A systematic review and meta-analysis protocol
Source: Medicine (Baltimore). 2018 Oct 12;97(41):e12654. doi: 10.1097/MD.0000000000012654 (PMC6203494; doi:10.1097/MD.0000000000012654)
Supplement: Supplemental Digital Content [file medi-97-e12654-s001.docx]

**Appendix 1. Search strategies (*MEDLINE (Ovid Online))***

1. exp Laparoscopy/

2. exp Surgical Procedures, Minimally Invasive/

3. exp Video-Assisted Surgery/

4. (laparoscop* or coelioscop* or celioscop* or peritoneoscop* or minimally invasive or video assisted surgery).mp.

5. OR/1-4

6. exp Pain, Postoperative/

7. exp Analgesia/

8. exp Pain management/

9. exp Analgesia, Patient-controlled/

10. (pain* or analgesi* or ache* or suffering* or discomfort).mp.

11. OR/6-10

12. exp Acupuncture/

13. exp Acupuncture therapy/

14. exp Electroacupuncture/

15. exp Acupuncture points/

16. exp Meridians/

17. (acupuncture or electroacupuncture or electro-acupuncture or auriculoacupuncture or auriculo-acupuncture or dry needling or acupuncturist* or acupoint* or meridian*).mp.

18. OR/12-17

19. 5 AND 11 AND 18
